# Approaches and Criteria for Provenance in Biomedical Data Sets and Workflows: Protocol for a Scoping Review

**DOI:** 10.2196/31750

**Published:** 2021-11-22

**Authors:** Kerstin Gierend, Frank Krüger, Dagmar Waltemath, Maximilian Fünfgeld, Thomas Ganslandt, Atinkut Alamirrew Zeleke

**Affiliations:** 1 Department of Biomedical Informatics at the Center for Preventive Medicine and Digital Health Medical Faculty Mannheim Heidelberg University Mannheim Germany; 2 Department of Communications Engineering University of Rostock Rostock Germany; 3 Department of Medical Informatics, Institute for Community Medicine University Medicine Greifswald Greifswald Germany

**Keywords:** provenance, biomedical, workflow, data sharing, lineage, scoping review, data genesis, scientific data, digital objects, healthcare data

## Abstract

**Background:**

Provenance supports the understanding of data genesis, and it is a key factor to ensure the trustworthiness of digital objects containing (sensitive) scientific data. Provenance information contributes to a better understanding of scientific results and fosters collaboration on existing data as well as data sharing. This encompasses defining comprehensive concepts and standards for transparency and traceability, reproducibility, validity, and quality assurance during clinical and scientific data workflows and research.

**Objective:**

The aim of this scoping review is to investigate existing evidence regarding approaches and criteria for provenance tracking as well as disclosing current knowledge gaps in the biomedical domain. This review covers modeling aspects as well as metadata frameworks for meaningful and usable provenance information during creation, collection, and processing of (sensitive) scientific biomedical data. This review also covers the examination of quality aspects of provenance criteria.

**Methods:**

This scoping review will follow the methodological framework by Arksey and O'Malley. Relevant publications will be obtained by querying PubMed and Web of Science. All papers in English language will be included, published between January 1, 2006 and March 23, 2021. Data retrieval will be accompanied by manual search for grey literature. Potential publications will then be exported into a reference management software, and duplicates will be removed. Afterwards, the obtained set of papers will be transferred into a systematic review management tool. All publications will be screened, extracted, and analyzed: title and abstract screening will be carried out by 4 independent reviewers. Majority vote is required for consent to eligibility of papers based on the defined inclusion and exclusion criteria. Full-text reading will be performed independently by 2 reviewers and in the last step, key information will be extracted on a pretested template. If agreement cannot be reached, the conflict will be resolved by a domain expert. Charted data will be analyzed by categorizing and summarizing the individual data items based on the research questions. Tabular or graphical overviews will be given, if applicable.

**Results:**

The reporting follows the extension of the Preferred Reporting Items for Systematic reviews and Meta-Analyses statements for Scoping Reviews. Electronic database searches in PubMed and Web of Science resulted in 469 matches after deduplication. As of September 2021, the scoping review is in the full-text screening stage. The data extraction using the pretested charting template will follow the full-text screening stage. We expect the scoping review report to be completed by February 2022.

**Conclusions:**

Information about the origin of healthcare data has a major impact on the quality and the reusability of scientific results as well as follow-up activities. This protocol outlines plans for a scoping review that will provide information about current approaches, challenges, or knowledge gaps with provenance tracking in biomedical sciences.

**International Registered Report Identifier (IRRID):**

DERR1-10.2196/31750

## Introduction

The (re-)use of electronic medical and patient-related data offers enormous potential for further investigations in clinical research [1,2]. Different national initiatives such as the French Health Data Hub initiative or the German Medical Informatics Initiatives are committed to better knowledge discovery and data sharing in the health care domain [3]. Resulting outcomes enable patients and physicians a safe and rapid access to therapies or treatment options. Subsequently, treatment costs can be reduced. In this context, the access to quality-assured, traceable, and hence, credible shared data is essential. Providing information about the origin of data demands concepts for traceability to gain understanding for the relationships between results and source data. There is an increasing interest and need to ensure traceability throughout scientific practice. Consequently, a systematic knowledge compilation regarding provenance and potential gaps is needed.

Provenance describes the origin of data. A basic understanding of the term “provenance” is given with the description “what happened” to the data [4]. Several different models exist to formally express provenance information, for instance, the World Wide Web Consortium PROV standard or CWLProv [5,6]. Advantages and opportunities of providing data provenance have been demonstrated, for instance, from the experiences in the EU-Horizon 2020 TRANSFoRm project [4]. Moreover, the importance of provenance and the relation to provenance within electronic health records is pointed out in the study of Johnson et al [7]. A previously published systematic review of provenance systems already investigated tools and systems [8]. However, our own work aims to understand current approaches and criteria as well as knowledge gaps for provenance in biomedical as well as domain-independent research.

The fields of research data management and FAIR (findable-accessible-interoperable-reusable) data principles consider provenance as one of the research pillars [9]. As such, a provenance-oriented approach requires thorough planning, execution, and evaluation of data management processes in the respective application domain [1]. While capturing provenance information in the research, adherence to criteria such as consistency, interoperability, and confidentiality are required across all software tools [2]. Furthermore, data privacy issues have to be respected during modeling to keep compliance with national and international requirements such as the European General Data Protection Regulation [10,11].

Process quality with the associated workflow quality can be achieved by monitoring and troubleshooting in applications or in data integration scenarios such as Extract-Transform-Load jobs. This implies workflow requirements to be established on a fine- or coarse-grained provenance level for troubleshooting [12]. Addressing data quality issues should support in reaching completeness, accuracy, and timeliness of the data and creates trust in it. However, heterogeneous data sources, dynamic infrastructures, data exchange across boundaries, and lack of standards for quality measures characterize the current state of electronic health record data sets [13]. Contrarily, provenance information strengthens the credibility of the data and proves that data have not been intentionally or unintentionally changed in its life cycle [14]. The concept and implementation of provenance is essential in most scientific domains such as environmental fields (geoprocessing workflows or climate assessments), in fusion engineering, or material sciences [15,16]. Since the use of machine learning techniques within the scope of decision support is becoming increasingly popular for medical researchers, they are under the obligation to prove their reproducibility [17]. Therefore, systematic knowledge about the “what happened” and about reproducibility metrics such as data sets and code accessibility is indispensable and is in need of further investigation to provide provenance [18].

The aim of this scoping review is to investigate existing evidence regarding approaches and criteria for provenance tracking as well as disclosing current knowledge gaps in the biomedical domain. This comprises modeling aspects as well as metadata frameworks for meaningful and usable provenance information during creation, collection, and processing of (sensitive) scientific biomedical data. The review also covers the examination of quality aspects of provenance criteria.

## Methods

### Design

The individual elements from the framework of Arksey and O’Malley [19] will be used as a roadmap for this scoping review. Essential methodological steps will cover the stages (1) identification of the research questions, (2) identification of relevant studies, (3) study selection, (4) data extraction and charting, and (5) collating, summarizing, and reporting the results. Any subsequent deviations of the final report from the scoping review protocol will be clearly highlighted and explained in the scoping review report.

### Ethics

Ethical approval was not required because only literature will be evaluated without processing sensitive patient data.

### Stage 1: Identification of the Research Questions

At first, an informal prescreening of relevant literature in PubMed and Web of Science as well as grey literature from conferences or organizations was carried out to determine the keywords in scope. Relevant literature was identified with the support of a librarian. PubMed was searched using the keywords “provenance” and “tracking.” The reviewer team explored, studied, and scrutinized additional literature based on search combinations of terms linked to the topic “provenance.” Ten publications were selected and reviewed by the team in an iterative process to guide the implementation of the research questions. During this step, keywords from titles and abstracts were gathered and analyzed by implementing the search strategy based on them. The following research questions were generated to meet the objective of this scoping review before study conduction: to investigate existing evidence regarding approaches and criteria for provenance tracking as well as disclosing current knowledge gaps in the biomedical domain. This review covers modeling aspects as well as metadata frameworks for meaningful and usable provenance information during creation, collection, and processing of (sensitive) scientific biomedical data. This review also covers the examination of quality aspects of provenance criteria.

Research question 1: Which potential (methodological) approaches exist for the classification and tracking of provenance criteria and methods in a biomedical or domain-independent context?

Research question 2: How can the potential value of provenance information be harnessed and by whom? How can usability be provided?

Research question 3: What are the challenges and potential problems or bottlenecks for the accomplishment of provenance?

Research question 4: Which guidelines or demands for the consideration of provenance criteria in a biomedical or domain-independent context have to be followed?

Research question 5: How completely can provenance be mapped in the data lifecycle or during data management?

### Stage 2: Identification of Relevant Studies

Relevant publications will be retrieved using concepts together with their associated keywords as selected from “Stage 1: Identification of the research questions.” Concepts are categorized into 4 groups: target domain, provenance, provenance properties, and objective. Target domain refers to the context of the research topic and includes studies with a biomedical, health care, clinical, or scientific background. Scientific background is limited to domain-independent studies and excludes all other domain-specific studies. The concept “provenance” concerns the information about the genesis of a given object while the concept “provenance properties” covers specific requirements tied to the term “provenance” or describes selected characteristics in this context. The concept “objective” embraces the range of purpose or the intention of provenance. Table 1 provides an overview of the eligibility criteria derived from the categorization of the concepts together with the defined terms and their matching keywords.

**Table 1:** Concepts and matching keywords (eligibility criteria).

Concepts	Matching keywords (inclusion criteria)
Target domain	biomed*^a^, EHR, electronic health record, healthcare, clinical, scientific^b^
Provenance	provenance, prov, lineage
Provenance properties	interop*, (data NEAR/2 [flow, quality, transformation]), metadata, workflow, semantic, framework, annotat*, ontolog*, management, document*, (model NEAR/2 provenance)
Objective	audit*, decision support, ETL, Extract-Transform-Load, FHIR, record linking, machine learning, reproducib*, transparen*, track*, implement*

^a^The * symbol (wildcard character) replaces or represents one or more characters.

^b^Will be used in a domain-independent context only.

A comprehensive search strategy for identifying the relevant literature, based on the given table, was implemented in PubMed and Web of Science. Medical subject headings were applied in PubMed. Additionally, the Boolean operators AND OR were used within the search strategy for combining the individual concepts and their associated keywords.

The inclusion criteria comprised all papers in the English language and published between January 1, 2006 and March 23, 2021. The concepts and their related keywords, as shown in Table 1, are considered during the selection of the papers within the biomedical or domain-independent area. The start date for inclusion of literature was chosen owing to the initiation of the Open Provenance Model in 2006 as a result of the Provenance Challenge series [20]. Grey literature from relevant project reports and proceedings were searched and reviewed for eligibility. All search results were exported to a reference management tool to eliminate duplications. Unique results were exported to the web-based screening tool Rayyan (Qatar Computing Research Institute) [21]. The PRISMA-ScR (Preferred Reporting Items for Systematic reviews and Meta-analyses extension for Scoping Reviews) will be used for reporting of this scoping review [22].

### Stage 3: Study Selection

During the scoping review process, decisions to select or eliminate studies are tracked using Rayyan. That way, independent screening by the reviewers is enabled. Rayyan allows citation sharing and blinded comparison of decisions for inclusion and exclusion of selected studies. All imported publications will be screened by reading the title and abstract by all 4 reviewers. Title-abstract screening is the process of reviewing the references for inclusion based solely upon their title and abstract. Reviewers will screen out irrelevant references whereby the inclusion and exclusion criteria serve as the basis for their eligibility decision. Conflicts will be resolved since at least 3 unified classifications are necessary for inclusion or exclusion of a publication in an unblinded modus. The included (=eligible) publications will be examined in a full-text screening phase to determine the extent to which they can answer the research questions. Each publication must be read by 2 researchers to determine the relevance to the research questions. If there is no joint agreement, an independent researcher will be consulted. A description and a PRISMA flow chart of the selection process with frequencies for references considered in the different databases will be provided as well as counting in the subsequent title-abstract screening process based on the eligibility criteria.

### Stage 4: Data Extraction and Charting

The data collection process will be documented by the reviewers while using the collectively developed template as provided in Table 2. The approach to data extraction needs to be consistent with the research question and purpose. This charting form will be pretested and will be used after closed alignment between the reviewers. “Pretested” means that 2 reviewers will independently complete the template for 5 studies ahead of the main study. They will compare the result with regard to a consistent approach and agree on necessary updates in the template, if necessary. Reviewers will diligently extract and update the study data from the identified papers in scope during their full-text review in an iterative process.

**Table 2:** Data charting template for key information from eligible papers.

Metadata publication	Characteristic extraction and specification
Title^a^	Title
Citation details^a^	Author (1st), journal, DOI
Year of publication^a^	For example, YYYY
Publication type^a^	Journal or website or conference, etc
Study type^a^	Use case or development or evaluation
Continent of study	For example, Australia
Institute^a^	Contributing institute (corresponding author or—if not provided—1st author)
Corresponding author’s discipline	For example, data architect
Funding source	Public or industry or none or missing
Objective^a^	Aim of the publication
Methods	Strategies, processes, or techniques utilized in the collection or analyzing of data, how is the validity of the study judged
Summary results^a^	Short description of results
Conclusion	Short description of conclusion
Target domain^a^	Name specific domain or domain independent
Keywords	List keywords from abstract
Metadata to key findings related to research questions	Characteristic extraction and specification
Research question 1: Approaches for classification and tracking of provenance criteria and methods in biomedical or domain-independent context	Provide description in the domain for data suitability or data availability and other requirements or factors on data or systems regarding the trace of the data history (eg, role of provenance in terms of domain standards, ie, interoperability standards, FAIR [findable-accessible-interoperable-reusable] data, relation to metadata and model use, representation formalisms, etc), check definition of provenance
Research question 2: Potential value of provenance information	Provide possible use case description and types of data sources included, usability including effect on target domain and by whom it can be used and who will be the stakeholders; problems, if provenance is not available
Research question 3: Potential problems or bottlenecks for the accomplishment of provenance	Describe any challenges (eg, legal, organizational, or technical conditions) or problems that occurred during implementation phase of provenance
Research question 4: Guidelines or demands for the consideration of provenance to be adhered to	Describe any valid domain standard requirement, for example, legal, guidelines, rules
Research question 5: Completeness of provenance information during data management process or data life cycle	Describe any measurement or outcome available for completeness of provenance information

^a^Obligatory input.

### Stage 5: Collating, Summarizing, and Reporting the Results

The charting results from stage 4 will be presented in the following steps [19]. Analysis will be given by a qualitative evaluation and by summary statistics, charts, or equivalent appraisal. The reporting of the results and outcome will be aligned to the research questions. The meaning of the findings and their relation to the overall objectives will be discussed. Implications for future research, practice, and policy will be outlined. The reporting of the results will be aligned with the PRISMA-ScR reporting guidelines [22].

## Results

### Schedule

The scoping review started with a tentative search of the databases in PubMed and Web of Science in early 2021 (see stages 1-3) and resulted in 469 matches. These papers will be subjected to title-abstract screening in an interactive selection process for eligibility, followed by a full-text screening stage. These papers will be examined within an iterative selection process for inclusion into data charting (see stage 4). Data extraction will be finalized during the 4th quarter of 2021. The scoping review will be completed by summarizing and synthesizing the results by February 2022 (see stage 5).

### Anticipated Outcomes

The scoping review will identify potentially relevant initiatives on provenance, and it will provide an overview of the evidence, gaps, and limitations for provenance criteria. All the evidence will be elaborated on the basis of the research questions. As such, the review can serve as preparatory work for achieving a comprehensive usable result on approaches and criteria for provenance. Based on the review results, the quality of the provenance criteria will be examined for a potential demarcation regarding minimum requirements for structuredness and completeness of provenance. We believe that this investigation supports provenance research with respect to the implementation of provenance in secondary use projects such as the German Medical Informatics Initiative. Within the Medical Informatics in Research and Care in University Medicine consortium, as part of the Medical Informatics Initiative, provenance has an important meaning to bioinformaticians and researchers [23].

## Discussion

Implications for future work will be derived from the current status of research activities and their underlying concepts. We anticipate that implications will encompass conceptual and modeling approaches up to the generation of provenance-aware data as well as gaps in the current practices within the health care domain. We believe that our results will support the further development of guidelines, thereby overcoming the identified challenges and disclosing new opportunities for the classification and tracking of provenance criteria. Evidence will assist in recognizing and defining the preconditions for data sharing. It will further characterize data suitability and categories (eg, data governance, relevance, quality) at a fitness for purpose level in the health domain, considering the interests of different stakeholders. Finally, the scoping review will provide insights into whether a further assessment of the results is useful within a full systematic review.

## Abbreviations

PRISMA-ScR: Preferred Reporting Items for Systematic reviews and Meta-Analyses extension for Scoping Reviews
